# Hepatic drug-metabolizing enzymes and drug transporters in Wilson’s disease patients with liver failure

**DOI:** 10.1007/s43440-021-00290-8

**Published:** 2021-06-11

**Authors:** Sylwia Szeląg-Pieniek, Stefan Oswald, Mariola Post, Joanna Łapczuk-Romańska, Marek Droździk, Mateusz Kurzawski

**Affiliations:** 1 Department of Experimental and Clinical Pharmacology, Pomeranian Medical University, Al. Powstańców Wlkp. 72, 70-111, Szczecin, Poland; 2 Institute of Pharmacology and Toxicology, Rostock University Medical Center, 18051 Rostock, Germany; 3 Department of General and Transplantation Surgery, Marie Curie Hospital, Arkońska 4, 71-455 Szczecin, Poland

**Keywords:** CYP450 enzymes, Drug transporters, Wilson’s disease, Protein abundance, Liver

## Abstract

**Background:**

Wilson’s disease is a genetic disorder inherited in a recessive manner, caused by mutations in the copper-transporter ATP7B. Although it is a well-known disease, currently available treatments are far from satisfactory and their efficacy varies in individual patients. Due to the lack of information about drug-metabolizing enzymes and drug transporters profile in Wilson’s disease livers, we aimed to evaluate the mRNA expression and protein abundance of selected enzymes and drug transporters in this liver disorder.

**Methods:**

We analyzed gene expression (qPCR) and protein abundance (LC–MS/MS) of 14 drug-metabolizing enzymes and 16 drug transporters in hepatic tissue from Wilson’s disease patients with liver failure (*n* = 7, Child–Pugh class B and C) and metastatic control livers (*n* = 20).

**Results:**

In presented work, we demonstrated a downregulation of majority of CYP450 and UGT enzymes. Gene expression of analyzed enzymes ranged between 18 and 65% compared to control group and significantly lower protein content of CYP1A1, CYP1A2, CYP2C8, CYP2C9, CYP3A4 and CYP3A5 enzymes was observed in Wilson’s disease. Moreover, a general decrease in hepatocellular uptake carriers from SLC superfamily (significant at protein level for NTCP and OATP2B1) was observed. As for ABC transporters, the protein abundance of BSEP and MRP2 was significantly lower, while levels of P-gp and MRP4 transporters were significantly higher in Wilson’s disease.

**Conclusions:**

Altered hepatic expression of drug‐metabolizing enzymes and drug transporters in Wilson’s disease patients with liver failure may result in changes of drug pharmacokinetics in that group of patients.

**Supplementary Information:**

The online version contains supplementary material available at 10.1007/s43440-021-00290-8.

## Introduction

Liver is a major organ involved in a number of key metabolic processes, which have impact on all body systems. It plays a central role in transformation of endogenous substrates i.e. sterols, fatty acids and eicosanoids, as well as it is involved in xenobiotics detoxification, including drug biotransformation [1]. The main components involved in those processes are intracellular drug-metabolizing enzymes and membrane drug transporters.

Drug metabolism in the liver is carried out by diverse groups of enzymes engaged in various reactions, including oxidation, reduction, hydrolysis or conjugation that results in biotransformation of pharmaceutical compounds. A large group of cytochrome P450 enzymes (CYP450), involved in oxidative reactions of I phase of drug biotransformation, plays a main role in that process. CYP superfamily is represented by 17 families and 39 subfamilies, and many of CYP enzymes show the highest abundance in the human liver. The most important hepatic monooxygenases for drug pharmacokinetics in humans are CYP1A2, CYP2A6, CYP2B6, CYP2C8, CYP2C9, CYP2C19, CYP2D6, CYP2E1, CYP3A4 and CYP3A5. In the phase II of drug metabolism, conjugation reactions are crucial, that allow to generate more soluble products, which can be easily excreted into bile or urine. UDP glycosyltransferases (UGTs) play a major role in phase II biotransformation, taking part in glucuronidation process [2, 3].

Drug transporters are membrane proteins, which govern transmembrane movement of drug molecules, other xenobiotics and endogenous substrates. They are classified into two superfamilies: ATP-binding cassette transporters (ABC) and solute carriers (SLC). ABC transporters utilize ATP as an energy source for transport of substrates against the concentration gradient. There are 7 subfamilies of ABC transporters, and three of them are of greatest importance for hepatic drug transport: ABCB, ABCC and ABCG. SLC carriers belong to a large protein family, represented by more than 400 membrane transporters, classified into 65 subfamilies. Both ABC transporters and SLC carriers are involved in both cellular efflux and influx of drug molecules and variety of endogenous substrates [4, 5].

A great variation in cytochrome P450 enzymes and drug transporters has been reported in the human liver, both at mRNA and protein level. Altered activity of drug-metabolizing enzymes and transporting proteins mediate responses to physiological and pathological stimuli, and was also documented in various liver diseases, and altered drug pharmacokinetics was documented in the organ pathology [6–10]. The major cause of liver damage worldwide is alcohol abuse and viral hepatitis that sometimes lead to irreversible liver failure and a need for liver transplantation [11–13]. Less common causes include autoimmune and genetic diseases [14, 15], drug-induced liver injury [16] and non-alcoholic fatty liver disease [17].

Wilson’s disease (WD) is a rare inherited monogenic disorder associated with pathological copper deposition in various body tissues. The prevalence of WD is relatively rare, with a frequency estimated to be 1 in ~ 30,000 [18].The disease is caused by mutations in the *ATP7B* gene, leading to disturbance in copper efflux from the liver and copper accumulation in tissues. Due to the copper retention in such organs like the liver, brain, and eyes, the most common symptoms of Wilson’s disease are hepatic and neurologic manifestations. Although the etiology of WD is well-known, the disease shows heterogeneous course in individual patients. Wilson’s disease patients differ in terms of age at the WD onset, as well as the first manifestation signs. Moreover, the severity of the symptoms varies between patients [19]. Currently, only symptomatic treatment is available, which include lifelong administration of copper-chelating medications, and the efficacy of therapy varies among individual patients. In case of insufficiency of available medication or acute liver failure, the only available method of treatment is liver transplantation (LT). It is estimated, that LT due to Wilson’s disease account for less than 1% of all LTs performed. However, approximately five percent of Wilson’s disease patients may experience acute liver failure resulting in a need for LT [15, 20].

Apart from treatment aiming at reduction of copper content in body, WD patients are often administered drugs for neurological and psychiatric symptoms, muscle tremors, as well as various comorbid diseases. Obviously, pharmacokinetics of those drugs may be influenced by altered hepatic drug metabolism and transport. To the best of our knowledge, there are no comprehensive data on drug-metabolizing enzymes and drug transporters in the liver of WD patients. Therefore, the aim of this study was to evaluate the gene expression and protein abundance of clinically relevant enzymes and transporters involved in drug biotransformation and transport in livers of patients suffering from liver failure due to Wilson’s disease.

## Materials and methods

### Tissue collection

The liver tissue specimens were obtained from 7 Caucasian patients with Wilson’s disease (4 male, 3 female), aged 20–46 (33 ± 9 years). All patients met clinical criteria for liver transplantation and represented the Child–Pugh class score B or C. The liver samples from Wilson’s disease patients were dissected from liver parenchymal tissue during elective liver transplantation.

The control samples were taken from non-tumorous liver tissue of patients undergoing resection of metastatic tumors, as described earlier [10]. The patient characteristics is presented in Table 1. The data of *ABCB1*, *ABCC1*, *ABCC2*, *ABCC3*, *ABCC4*, *ABCG2*, *ABCB11*, *SLC10A1*, *SLC22A1*, *SLC22A3*, *SLC22A7*, *SLCO1B1*, *SLCO1B3* and *SLCO2B1* transporters for control samples have been published earlier in [10] and used in current manuscript for comparison purpose.

**Table 1:** Characteristics of the subjects (mean ± SD)

Parameter/disease	Controls *n* = 20	WD *n* = 7
Sex (male/female)	11/9	4/3
Age (years)	63 ± 10	33 ± 9
Child–Pugh (B/C)	–	2/5
Total bilirubin (mg/dl)	0.59 ± 0.25	24.2 ± 31.9
Albumin (g/dl)	3.89 ± 0.38	3.2 ± 0.6
PT (s)	12.7 ± 2.3	34.5 ± 15.4
INR	1.14 ± 0.21	3.6 ± 1.8

*WD* Wilson’s disease, *PT* prothrombin time, *INR* international normalized ratio

Resected tissues samples were immediately snap-frozen in liquid nitrogen for protein analysis or stored in RNAlater solution (Applied Biosystems, Darmstadt, Germany) for RNA analysis. All samples were stored at − 80 °C until further processing. The study protocol was approved by the local Bioethics Committee at Pomeranian Medical University, Szczecin, Poland.

### mRNA isolation and quantification

Total RNA was isolated from 25 mg of the liver sample using Direct-zol RNA MiniPrep kit (Zymo Research, USA). RNA concentration and purity was assessed using DS-11 FX spectrophotometer (Denovix, USA). cDNA synthesis was performed using SuperScript VILO Master Mix (Thermo Fisher Scientific, USA), with 500 ng of RNA for 20 ul of reaction volume, according to manufacturer instructions. The gene expression of 14 drug-metabolizing enzymes and 16 transporters was determined by means of real-time PCR, using TaqMan Fast Advanced Master Mix and pre-validated TaqMan assays (Thermo Fisher Scientific, USA). Details of TaqMan assays of the tested genes and reference controls are provided in Supplementary Table S1. Quantitative real-time PCR analysis was performed in a volume of 10 ul in ViiA 7 Real-Time PCR System (Life Technologies, USA). The gene expression levels were examined in duplicate, and mean CT (cycle of threshold) values were used for further analysis. Relative expression (relative quantity—RQ) of the analyzed genes was calculated using the ∆CT method—normalized to mean expression of the housekeeping genes (*GAPDH, GUSB, HMBS, PPIA, RPLP0* and *RPS9*) and presented in figures. Additionally, ∆∆CT values normalized to mean value for the control group were presented in tables (Supplementary Tables S2, S3).

### Protein quantification by LC–MS/MS

Protein quantification of biotransformation-related enzymes (CYP1A1, CYP1A2, CYP2B6, CYP2C8, CYP2C9, CYP2C19, CYP2D6, CYPE1, CYP3A4, CYP3A5, UGT1A1, UGT1A3, UGT2B7 and UGT2B15), ABC transporters (P-gp, MRP1, MRP2, MRP3, MRP4, BCRP, and BSEP) and SLC transporters (NTCP, MCT1, OCT1, OCT3, OAT2, IMPT1, OATP1B1, OATP1B3 and OATP2B1) were measured by mass spectrometry-based targeted proteomics using a validated LC–MS/MS method, as recently described [21].

In brief, about 40 mg of pulverized tissue was added to 1 ml lysis buffer (0.2% SDS, 5 mM EDTA) containing 5 µl/ml Protease Inhibitor Cocktail (ProteoExtract-Native Membrane Extraction Kit; Merck, Darmstadt, Germany), and manually homogenized using a Dounce homogenizer (10 strokes) before incubation for 30 min at 4 °C. After determination of the protein concentration (Pierce BCA Protein Assay Kit; Thermo Fisher Scientific, Hennigsdorf, Germany), a volume corresponding to 100 µg protein was subjected to the established method of filter-aided sample preparation, which generates tryptic digests of whole tissue lysates and avoids potential disadvantages of other sample preparation methods, such as sample loss or the enrichment of certain cell fractions [10]. The resulting protein data were normalized to the respective mass of tissue lysate used in the tryptic digest. LC–MS/MS analyses were conducted on API4000 triple quadrupole mass spectrometer (AB Sciex, Foster City, CA, USA) coupled to a Shimadzu LC (SLC-10A VP) system (Shimadzu, USA) and an HTS PAL LEAP autosampler (LEAP Technologies, USA). The details of the procedure, peptides used and mass transitions are described elsewhere [10, 21].

### Statistical analysis

Normality of quantitative variables distribution was determined by means of Shapiro–Wilk test. Due to significant deviation from normal distribution, differences between study groups (WD, *n* = 7 and controls, *n* = 20) were further evaluated by means of nonparametric Mann–Whitney *U* test. The calculations were based on all samples, substituting undetectable protein concentrations (lower or equal to 0.1 nmol/l, LLOQ) by zero. *p* values < 0.05 were considered to be statistically significant. The statistical calculations were performed using Statistica 13.3 Software Package (TIBCO Software Inc, Palo Alto, CA, USA).

## Results

### mRNA quantification and protein abundance of drug metabolizing enzymes

Our study showed that all of the tested enzyme and transporter genes were expressed at detectable levels (CT < 35) in all analyzed liver samples (Table S2). The protein abundance analysis revealed that the evaluated enzyme proteins were detected in almost all control samples (except CYP2D6 protein in one control and CYP1A1 protein in two controls) but not in all WD patients (Table S3). All the analyses were done in the same number of subjects (WD: *n* = 7 and controls: *n* = 20).

**Fig. 1:**
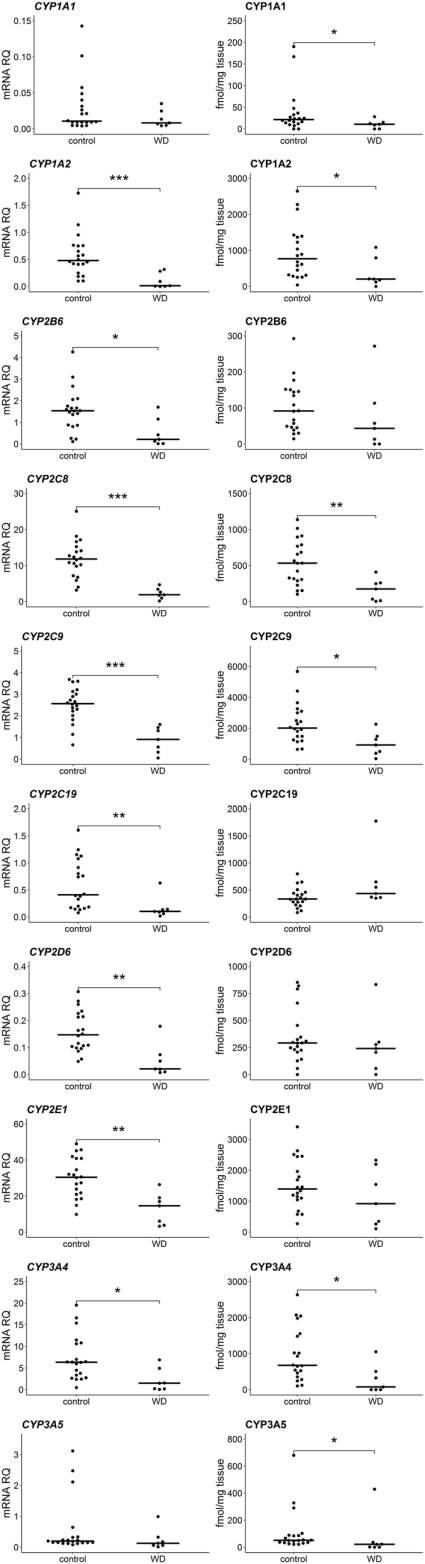
Gene expression (left) and protein abundance (right) of CYP450 enzymes in hepatic tissues from Wilson’s disease (WD, *n* = 7) patients and controls (*n* = 20). Horizontal bars represent median values for each group. mRNA level of the analyzed genes was expressed as a relative amount to the mean of the housekeeping genes (*GAPDH, GUSB, HMBS, PPIA, RPLP0, RPS9*). Statistically significant differences: *p < 0.05, **p < 0.01, ***p < 0.001 (Mann–Whitney *U* test) in comparison with the controls

**Fig. 2:**
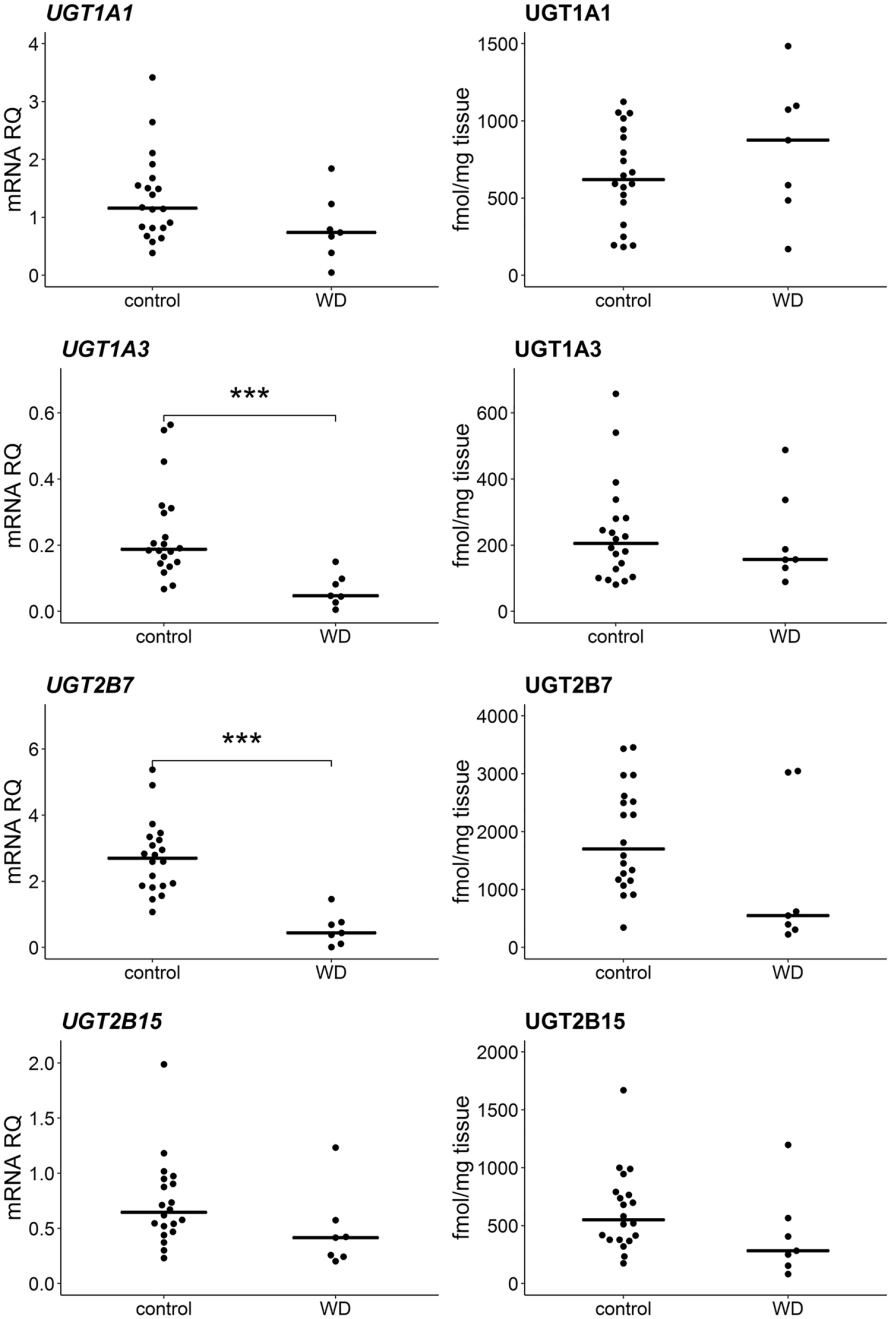
Gene expression (left) and protein abundance (right) of UGT enzymes in hepatic tissues from Wilson’s disease (WD, *n* = 7) patients and controls (*n* = 20). Horizontal bars represent median values for each group. mRNA level of the analyzed genes was expressed as a relative amount to the mean of the housekeeping genes (*GAPDH, GUSB, HMBS, PPIA, RPLP0, RPS9*). Statistically significant differences: *p < 0.05, **p < 0.01, ***p < 0.001 (Mann–Whitney *U* test) in comparison with the controls

We observed a markedly downward trend in the expression levels of all CYP450 genes and UGT enzymes in WD group, and it ranged between 18 and 65% compared to the control group. Statistically significant (Mann–Whitney *U* test, *p* < 0.05) downregulation of *CYP1A2* (*U* = 10; *N* = 27; *p* = 0.001), *CYP2B6* (*U* = 25; *N* = 27; *p* = 0.014), *CYP2C8* (*U* = 3; *N* = 27; *p* = 2 × 10^–4^), *CYP2C9* (*U* = 8; *N* = 27; *p* = 7 × 10^–4^), *CYP2C19* (*U* = 17; *N* = 27; *p* = 0.004), *CYP2D6* (*U* = 16; *N* = 27; *p* = 0.003), *CYP2E1* (U = 14; N = 27; p = 0.002), *CYP3A4* (*U* = 23; *N* = 27; *p* = 0.010), *UGT2B7* (*U* = 2; *N* = 27; *p* = 2 × 10^–4^) and *UGT1A3* (*U* = 10; *N* = 27; *p* = 0.001) at mRNA level was noted. Significantly, lower protein content in WD patients was observed for CYP1A1 (*U* = 34; *N* = 27; *p* = 0.049), CYP1A2 (*U* = 27; *N* = 27; *p* = 0.019), CYP2C8 (*U* = 19; *N* = 27; *p* = 0.005), CYP2C9 (*U* = 25; *N* = 27; *p* = 0.014), CYP3A4 (*U* = 24; *N* = 27; *p* = 0.012) and CYP3A5 (*U* = 30; *N* = 27; *p* = 0.029). The greatest decrease in protein abundances was documented for CYP1A1, CYP3A4 and CYP2C8, which were at 28%, 29% and 30% values compared to the control group, respectively. No significant differences were found among UGT proteins. Detailed data are presented in Figs. 1 and 2 and Supplementary Table S2 and S3. mRNA quantification results are additionally normalized to the mean value of the controls in the tables (∆∆CT method).

### mRNA quantification and protein abundance of drug transporters

**Fig. 3:**
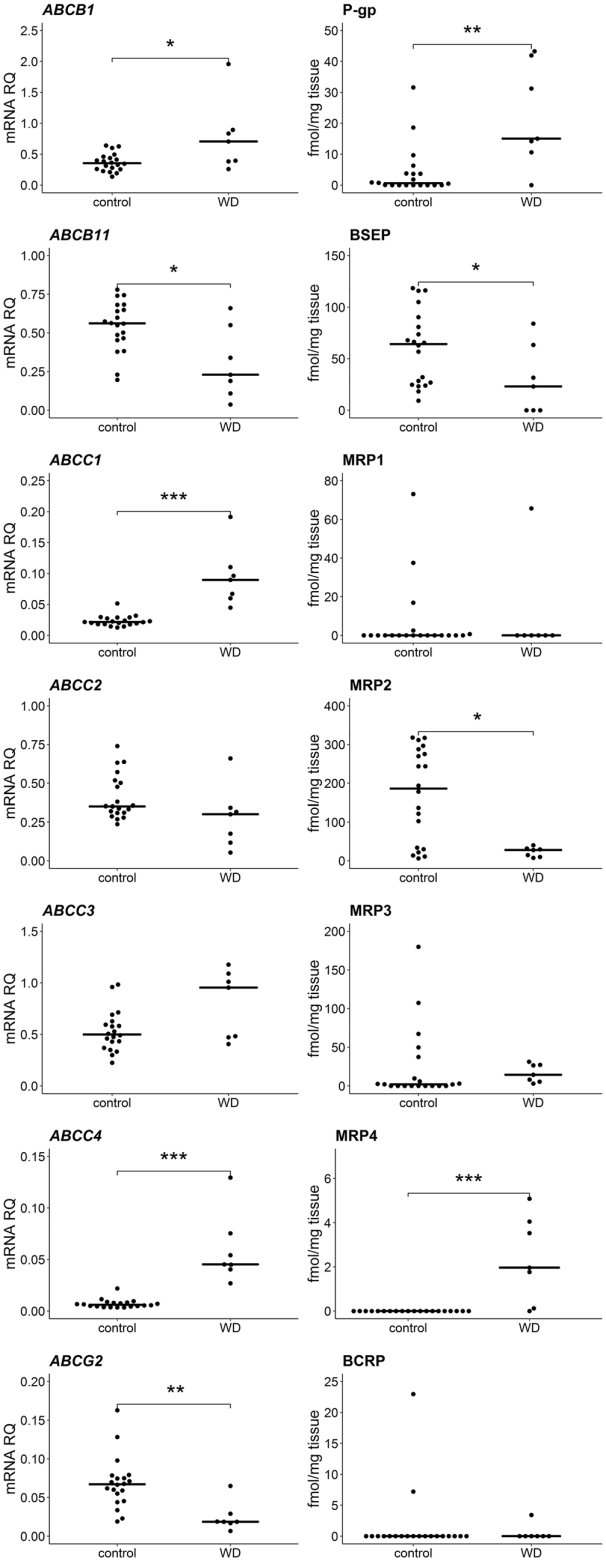
Gene expression (left) and protein abundance (right) of ABC transporters in hepatic tissues from Wilson’s disease (WD, *n* = 7) patients and controls (*n* = 20). Horizontal bars represent median values for each group. mRNA level of the analyzed genes was expressed as a relative amount to the mean of housekeeping genes (*GAPDH, GUSB, HMBS, PPIA, RPLP0, RPS9*). Statistically significant differences: *p < 0.05, **p < 0.01, ***p < 0.001 (Mann–Whitney *U* test) in comparison with the controls. The data for *ABCB1*, *ABCC1*, *ABCC2*, *ABCC3*, *ABCC4*, *ABCG2* and *ABCB11* transporters for control samples were published earlier in [10]

As for ABC transporters, the mRNA expression levels were significantly increased in WD group in case of *ABCB1* (*U* = 32; *N* = 27; *p* = 0.038), *ABCC1* (*U* = 1; *N* = 27; *p* = 2 × 10^–4^) and *ABCC4* (*U* = 0; *N* = 27; *p* = 1 × 10^–4^). A corresponding statistically significant increase of protein abundance was observed for P-gp and MRP4 (5.5-fold and 2.3-fold higher mean protein content in the controls, respectively), while MRP1 protein (encoded by *ABCC1* gene) was detected only in some individuals. Decreased mRNA expression in the livers from WD patients was documented for *ABCB11* (*U* = 27; *N* = 27; *p* = 0.019) and *ABCG2* (*U* = 11; *N* = 27; *p* = 0.001), while BSEP (encoded by *ABCB11* gene) and MRP2 (*ABCC2*) protein abundances were significantly lower [48% (*U* = 34; *N* = 27; *p* = 0.049) and 14% (*U* = 24; *N* = 27; *p* = 0.012)] of the mean value of the controls, respectively). BCRP (*ABCG2*) protein was quantified only in single individuals (Fig. 3, Supplementary Table S2, S3).

**Fig. 4:**
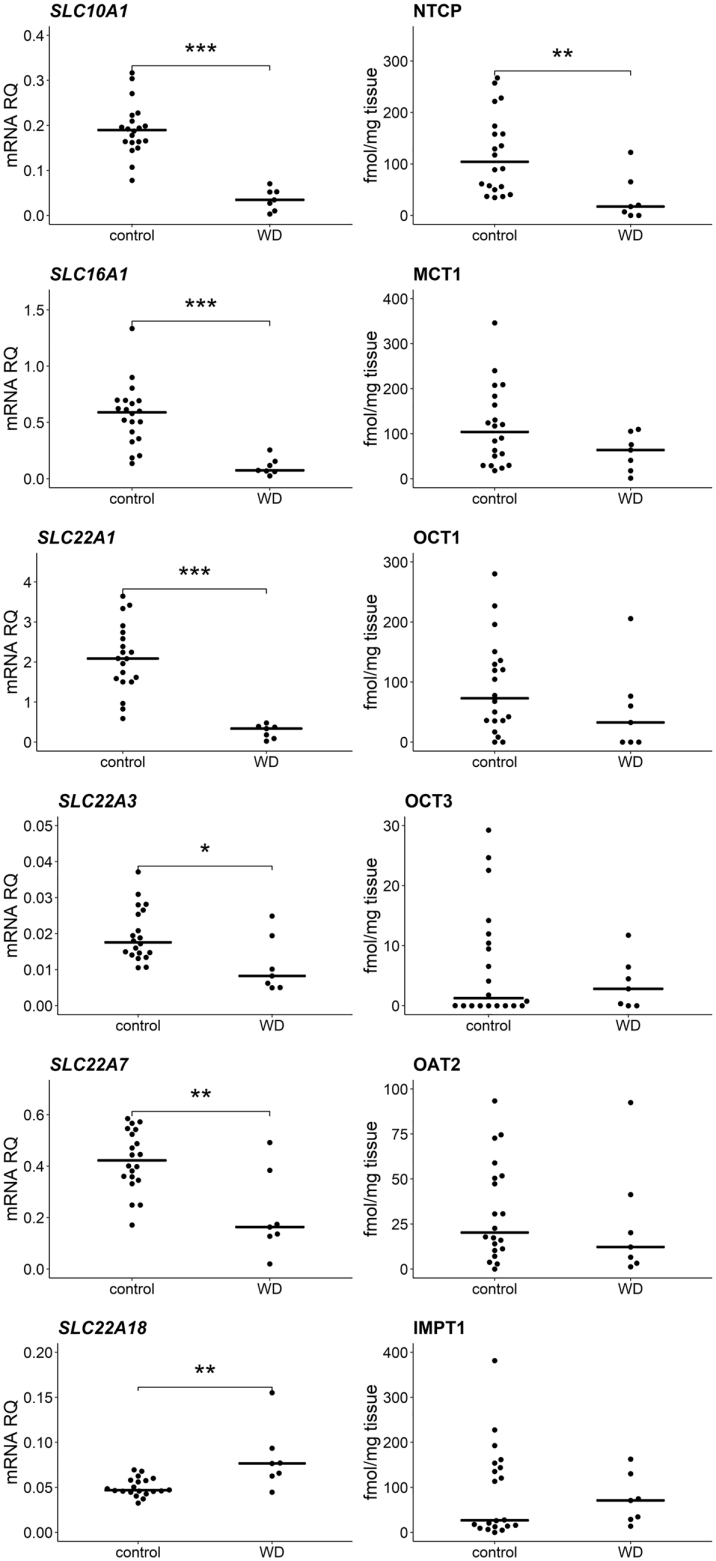
Gene expression (left) and protein abundance (right) of SLC carriers in hepatic tissues from Wilson’s disease (WD, *n* = 7) patients and controls (*n* = 20). Horizontal bars represent median values for each group. mRNA level of the analyzed genes was expressed as a relative amount to the mean of the housekeeping genes (*GAPDH, GUSB, HMBS, PPIA, RPLP0, RPS9*). Statistically significant differences: *p < 0.05, **p < 0.01, ***p < 0.001 (Mann–Whitney *U* test) in comparison with the controls. The data for *SLC10A1*, *SLC22A1*, *SLC22A3* and *SLC22A7* transporters for control samples were published earlier in [10]

**Fig. 5:**
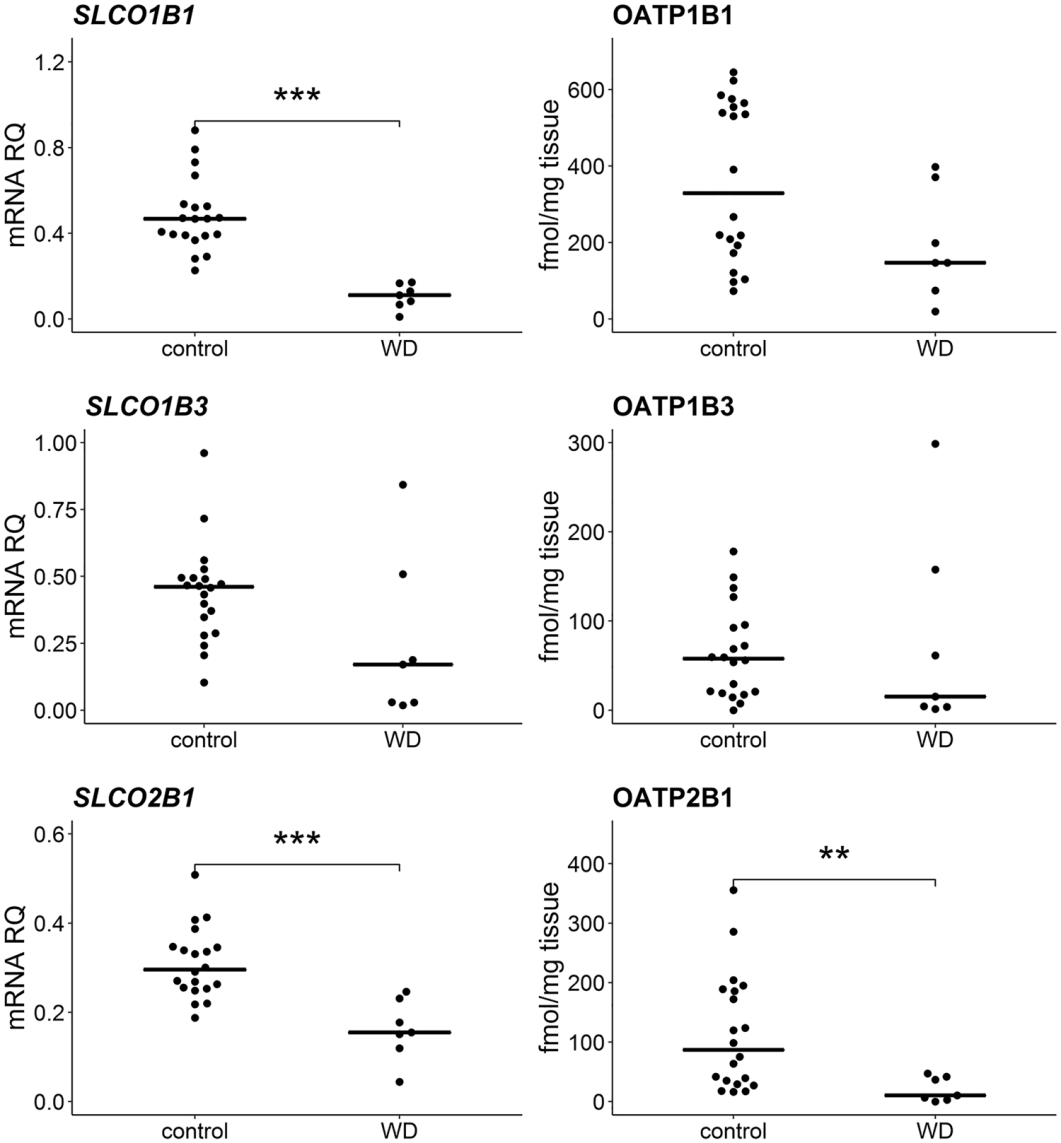
Gene expression (left) and protein abundance (right) of SLCO transporters in hepatic tissues from Wilson’s disease (WD, *n* = 7) patients and controls (*n* = 20). Horizontal bars represent median values for each group. mRNA level of the analyzed genes was expressed as a relative amount to the mean of housekeeping genes (*GAPDH, GUSB, HMBS, PPIA, RPLP0, RPS9*). Statistically significant differences: *p < 0.05, **p < 0.01, ***p < 0.001 (Mann–Whitney *U* test) in comparison with the controls. The data for *SLCO1B1*, *SLCO1B3* and *SLCO2B1* transporters for control samples were published earlier in [10]

Significant decrease in expression of 7 out of 9 analyzed SLC carriers was observed in WD [*SLC10A1* (*U* = 2; *N* = 27; *p* = 2 × 10^–4^), *SLC16A1* (*U* = 4; *N* = 27; *p* = 3 × 10^–4^), *SLC22A1* (*U* = 0; *N* = 27; *p* = 2 × 10^–4^), *SLC22A3* (*U* = 26; *N* = 27; *p* = 0.016), *SLC22A7* (*U* = 23; *N* = 27; *p* = 0.010), *SLCO1B1* (*U* = 0; *N* = 27; *p* = 2 × 10^–4^), *SLCO2B1* (*U* = 6; *N* = 27; *p* = 4 × 10^–4^)]. However, protein abundance was significantly lower only in case of NTCP (*SLC10A1*) (*U* = 19; *N* = 27; *p* = 0.005) and OATP2B1 (*SLCO2B1*) (*U* = 22; *N* = 27; *p* = 0.009), correspondingly with gene expression downregulations of these transporters. NTCP in WD group was observed to be at the level of 28% of the controls, whereas OATP2B1 showed approximately fivefold decrease. Unlike the remaining of the studied SLC transporters, *SLC22A18* was upregulated at mRNA level (*U* = 20, *N* = 27, *p* = 0.006), but significant change in the abundance of the encoded IMPT1 protein was not observed (Figs. 4, 5, Supplementary Tables S2, S3).

## Discussion

In the present study, we provided gene expression and quantitative protein analysis of 14 drug-metabolizing enzymes and 16 membrane transporters in livers derived from patients suffering from Wilson’s disease. To our knowledge, this is the first report providing a broad view on protein abundance and mRNA expression of genes related to drug metabolism and transport in that particular liver pathology.

Generally, our data on drug-metabolizing enzymes in Wilson’s disease end-stage livers are in the line with available data for other liver pathologies resulting in the organ failure. It is well documented, that hepatic activity and total content of cytochrome P450 enzymes are impaired in liver pathologies, and is generally downregulated in end-stage liver dysfunction [8, 22–26]. We reported a downregulation trend of the expression of most analyzed enzymes, as well as significant decrease in protein abundance of CYP1A1, CYP1A2, CYP2C8, CYP2C9, CYP3A4 and CYP3A5 in WD patients. Decrease in hepatic CYP1A2 content has been reported previously in other liver pathologies, i.e. non-alcoholic fatty liver disease (NAFLD), and at mRNA level—in alcoholic liver disease (ALD), primary sclerosing cholangitis (PSC) and hepatitis C virus (HCV)-induced liver damage. The downregulation of *CYP3A4* expression was also observed in HCV and ALD, but not in PSC and NAFLD, whereas *CYP2C9* showed no significant changes in the abovementioned pathologies [8, 22]. However, protein content of both abovementioned enzymes was significantly decreased in HCV and ALD [26]. In the case of CYP2B6, CYP2C19 and CYP2D6, we observed a significant decrease in mRNA levels, but not in protein abundance. It was previously reported that *CYP2B6* mRNA expression was decreased in hepatocellular carcinoma (HCC), and contrary, significantly increased in HBV and alcoholic cirrhosis, as well as in NAFLD [8, 22]. However, in Caucasians, no statistically significant changes in *CYP2B6* expression in alcoholic liver disease, as well as in hepatitis C virus-induced liver damage and primary sclerosing cholangitis, were observed [23]. CYP2D6 was not reported to be affected in non-cancerous liver diseases [8, 23], except for protein levels in HCV cirrhosis [26]. As for CYP2C19, its expression is generally decreased in HCC and other liver pathologies, but the available data are not consistent [22, 23, 27]. Significant decrease in expression of *CYP1A2*, *CYP2C8*, *CYP2C9*, *CYP2E1* and *CYP3A4* was reported by Ren et al. [28] in tumor tissues from patients with hepatocellular carcinoma, what is in line with our result for these genes in Wilson’s disease livers. However, that data refer to cancer tissue and cannot be directly compared with other liver pathologies.

Aside from phase I drug-metabolizing enzymes, we also analyzed the expression of four major UDP glucuronosyltransferases (UGT). Our findings reveled significant decrease in expression only of *UGT1A3* and *UGT2B7* genes, but not significantly affected protein abundance of all analyzed UGTs. The protein data are not in keeping with the study of Debinski et al. [29], who showed an upregulation of UGTs in human liver injury of different than WD origin based on immunohistochemistry staining, and thus limitations on the method as polyclonal antibody used in that study recognized multiple isoforms of UGT from both family 1 and family 2, what could explain discrepancy of the findings. Nevertheless, other reports based on mRNA level analysis are in agreement with our study and indicate the decrease in expression of UGT in liver pathologies [30]. It is worth to mention, that UGT1A1 protein abundance is decreased in Atp7b−/− mouse model of Wilson’s disease [31]. However, our study did not demonstrate protein abundance changes in WD, but showed only a downward trend in UGT1A1 mRNA without statistical significance. As for protein data of other UGT enzymes, UGT2B7 and UGT2B15 showed significant decrease in HCV and ALD livers [26]. In our work in WD, we observed a downward trend in these proteins levels in the liver, but not statistically significant. It may be a result of a small tested group comprising only 7 patients with Wilson’s disease or a different pathological background.

Decreased content of CYP450 enzymes in Wilson’s disease may affect both efficacy and toxicity of many drugs. It seems particularly important in the case of CYP3A4, which is involved in metabolism of about 50% of all pharmaceuticals, many of them used to control neurologic and psychiatric WD signs and symptoms, which occur in 40% (neurologic) and (10–25% psychiatric) WD patients. As many of drugs used to treat those symptoms, e.g. SSRIs, anticonvulsants, benzodiazepines or antipsychotics are also extensively metabolized by other CYP450 enzymes, it is possible that CYPs downregulation may interfere with drug pharmacokinetics in Wilson’s disease patients [32]. It may be important not only for treatment of patients with severe liver dysfunction, but also in case of those, who presents psychiatric symptoms of WD. In this group of patients, higher plasma drug concentration caused by dysregulation of CYPs may potentially increase the risk for drug toxicity. Moreover, it is possible that dysregulation of hepatic drug-metabolizing enzymes may directly affect Wilson’s disease treatment, e.g. penicillamine, a chelating agent administered to all diagnosed WD patients, which undergoes hepatic metabolism [33]. However, specific enzymes and transporters involved in that process have not been identified, but it could be assumed that altered status of drug-metabolizing enzymes and membrane transporters may potentially impact penicillamine metabolism and clinical responses.

We observed significant upregulation of two efflux transporters belonging to ABC superfamily, i.e. *ABCB1*/P-gp and *ABCC4*/MRP4. Similar results for P-gp and MRP4 were earlier found in many other pathologies of the liver, e.g. alcoholic liver disease, non-alcoholic fatty liver disease, primary biliary cirrhosis (PBC) and HCV [7, 9, 10, 22, 34]. Moreover, both transporters are upregulated in autoimmune hepatitis (AIH) [10]. Due to markedly higher levels of MRP4 in different liver pathologies, this transporter is considered to be a potential marker for liver disease and its upregulation may serve to protect hepatocytes from negative impact of potentially toxic compounds such as bile acids [35]. In turn, increased level of P-gp in the liver may translate into reduced hepatic exposure to many drugs, that are substrates of the transporter, including antibiotics, anticoagulants, anticonvulsants, opioids, proton pomp inhibitors, anticancer drugs and many more [36]. In case of *ABCC1* and *ABCG2*, we demonstrated an upregulation of these genes expression in WD livers. Nevertheless, proteins of both transporters were detected only in several controls and Wilson’s disease patients. This study also documented downregulation of *ABCB11 *and encoded BSEP transporter levels in Wilson’s disease subjects. The available other studies indicate the increase in *ABCB11 *levels in cholestatic diseases and, contrary, downregulation in HCV and ALD [10, 34]. Upregulation of ABCB11 in cholestasis is a possible mechanism protecting hepatocytes from bile acids accumulation, which is also related with simultaneous increase in other efflux transporters [7]. BSEP plays a vital role in bile salt secretion from hepatocytes to the bile and its downregulation may result in limited excretion of bile salts. When not compensated, it may lead bile acid toxicity, resulting in intensified inflammatory and fibrotic processes in the liver [37]. An inverse association between elevated hepatic copper concentration and BSEP expression in human liver tissue was previously reported. Wooton-Kee et al. [38] showed that liver samples with high Cu levels had decreased *ABCB11* mRNA expression, possibly due to impaired binding of FXR (farnesoid X receptor), RXR (retinoid X receptor) and LRH-1 (liver receptor homolog-1) nuclear receptors to *ABCB11* gene’s promoter. So, these studied provide functional evidence for downregulation of BSEP protein abundance, which may potentially affect drug pharmacokinetics, e.g. pravastatin being BSEP substrate [39]. Besides a decrease in BSEP level, we also observed a significant downregulation in MRP2 protein, which indicated the presence of significant disturbances in biliary excretion in WD patients [40].

Our study demonstrated lower abundance of transporter proteins providing hepatocellular uptake functions in Wilson’s disease, but the difference reached statistical significance only in case of NTCP (sodium-taurocholate co-transporting polypeptide). Similar changes in NTCP protein abundance have been previously reported in ALD and HCV [10, 34]. This observation suggests the existence of mechanisms protecting hepatocytes from toxic effects of exo- and endogenous compounds, for example, bile acids [41]. Other SLC family carriers with a disturbed expression in WD livers were *SLC16A1* and *SLC22A18*. These transporters are mainly engaged in lactate and lipid handling, respectively; therefore, their downregulation in Wilson’s disease may result in disturbances in respective pathways [42, 43]. For three studied OATP transporters, we showed a significant decrease in OATP2B1, both at mRNA level and protein abundance, and downregulation of *SLCO1B1* gene expression (but not OATP1B1 protein level). OATPs are largely involved in the uptake of bile acids, conjugated steroids, thyroid hormones and peptides, as well as numerous drugs (e.g. statins, sartans and antibiotics) [44–46]. Due to their broad range of transported substrates, decrease in OATPs function may affect many endogenous processes related to transported substrates, which may result from liver failure progression in Wilson’s disease.

Drug transporters changes, i.e. decreased uptake activity along with activation of efflux functions, in Wilson’s disease livers suggests a presence of protecting mechanisms, reducing a negative impact of endo- and exogenic compounds. However, due to intensified oxidative stress, inflammation and fibrotic processes during high copper level in liver tissue, all those compensation mechanisms may not be sufficient.

It is possible, that copper excess in Wilson’s disease may have a direct impact on many intracellular pathways, including nuclear receptor signaling. The significant impairment of nuclear receptors expression and function have been reported in WD livers and WD animal models, as well as in cell lines treated with copper [38, 47–49]. There is strong evidence, that copper overload in Wilson’s disease may induce decrease in FXR (farnesoid X receptor), RXR (retinoid X receptor) and LRH-1 (liver receptor homolog-1) [38]. The abovementioned nuclear receptors play an important role not only in regulation of bile acid, cholesterol, lipid and glucose homeostasis but also drug-metabolizing enzymes and transporters [50].

The major limitation of this study is a relatively small number of the analyzed samples, which is related with low prevalence of Wilson disease. However, it gives some insight into handling of drugs and some endogenous pathways. Another issue is related to the control samples used (normal tissue resected from livers of metastatic tumor patients), which may not fully represent physiological state. Due to a small number of patients studied, we were also not able to stratify samples according to the disease stage, since previous report suggested that expression levels of drug transporters could be related to a functional state of the organ in different liver pathologies [10] Additionally, difference in group size between controls and WD may potentially cause a bias. Facing ethical aspects and practical problems associated with administration of drugs/medicinal products, which provide no potential benefits to patients with severe liver disease, such data like those being the result of the present study enable construction of physiologically based-pharmacokinetic models (PB/PK) for better prediction of drug responses.

In conclusion, our study confirmed that most of I phase and II phase enzymes are downregulated in Wilson’s disease patients with liver failure (Child–Pugh class B and C), and it is generally consistent with the results of other liver disorders. We also observed a downregulation of several uptake transporters belonging to SLC superfamily, with simultaneous increase in P-gp and MRP4 abundance. These findings may suggest existence of adaptation mechanisms that protect the liver against the harmful effects of endogenous and exogenous entities (decreased uptake functions of SLC carriers and increased efflux activity provided by ABC efflux transporters). The altered activity of drug-metabolizing enzymes and membrane transporters may also affect drug pharmacokinetics.

## Supplementary Information

Below is the link to the electronic supplementary material.

Supplementary file1 (DOCX 15 kb)

Supplementary file2 (DOCX 24 kb)

Supplementary file3 (DOCX 26 kb)
