# Supplementary material for: Hepatic drug-metabolizing enzymes and drug transporters in Wilson’s disease patients with liver failure
Source: Pharmacol Rep. 2021 Jun 11;73(5):1427–38. doi: 10.1007/s43440-021-00290-8 (PMC8460590; doi:10.1007/s43440-021-00290-8)
Supplement: Supplementary file 1 — Supplementary file1 (DOCX 15 kb) [file 43440_2021_290_MOESM1_ESM.docx]

Supplementary Table 2. Gene expression quantity data of the drug-metabolizing enzymes and drug transporters in controls (n=20) and Wilson’s disease livers (n=7).

|  | mean | SD | CV% | median | min | max | P value |  |
| --- | --- | --- | --- | --- | --- | --- | --- | --- |
| RQ | | | | | | |  |  |
| ***CYP1A1*** | |  |  |  |  |  |  |  |
| Controls | | 1.00 | 1.19 | 119% | 0.37 | 0.14 | 4.96 | 0.333 |
| WD | | 0.49 | 0.37 | 77% | 0.29 | 0.15 | 1.22 |  |
| ***CYP1A2*** | |  |  |  |  |  |  |  |
| Controls | | 1.00 | 0.65 | 65% | 0.84 | 0.18 | 3.04 | 0.001 |
| WD | | 0.18 | 0.23 | 126% | 0.02 | 0.00 | 0.55 |  |
| ***CYP2B6*** | |  |  |  |  |  |  |  |
| Controls | | 1.00 | 0.60 | 60% | 0.98 | 0.07 | 2.72 | 0.014 |
| WD | | 0.33 | 0.39 | 116% | 0.13 | 0.01 | 1.09 |  |
| ***CYP2C8*** | |  |  |  |  |  |  |  |
| Controls | | 1.00 | 0.42 | 42% | 0.99 | 0.27 | 2.11 | 2*10^-4^ |
| WD | | 0.19 | 0.12 | 63% | 0.16 | 0.01 | 0.39 |  |
| ***CYP2C9*** | |  |  |  |  |  |  |  |
| Controls | | 1.00 | 0.30 | 30% | 1.02 | 0.26 | 1.47 | 7*10^-4^ |
| WD | | 0.35 | 0.22 | 62% | 0.36 | 0.02 | 0.64 |  |
| ***CYP2C19*** | |  |  |  |  |  |  |  |
| Controls | | 1.00 | 0.73 | 73% | 0.68 | 0.13 | 2.67 | 0.004 |
| WD | | 0.28 | 0.32 | 114% | 0.17 | 0.03 | 1.05 |  |
| ***CYP2D6*** | |  |  |  |  |  |  |  |
| Controls | | 1.00 | 0.45 | 45% | 0.93 | 0.31 | 1.94 | 0.003 |
| WD | | 0.32 | 0.36 | 110% | 0.13 | 0.05 | 1.13 |  |
| ***CYP2E1*** | |  |  |  |  |  |  |  |
| Controls | | 1.00 | 0.35 | 35% | 1.01 | 0.32 | 1.62 | 0.002 |
| WD | | 0.43 | 0.27 | 63% | 0.48 | 0.11 | 0.87 |  |
| ***CYP3A4*** | |  |  |  |  |  |  |  |
| Controls | | 1.00 | 0.69 | 69% | 0.87 | 0.06 | 2.69 | 0.010 |
| WD | | 0.30 | 0.34 | 113% | 0.21 | 0.01 | 0.95 |  |
| ***CYP3A5*** | |  |  |  |  |  |  |  |
| Controls | | 1.00 | 1.50 | 150% | 0.35 | 0.14 | 5.54 | 0.175 |
| WD | | 0.44 | 0.56 | 127% | 0.22 | 0.03 | 1.76 |  |
| ***UGT1A1*** | |  |  |  |  |  |  |  |
| Controls | | 1.00 | 0.53 | 53% | 0.86 | 0.29 | 2.55 | 0.092 |
| WD | | 0.61 | 0.40 | 66% | 0.55 | 0.03 | 1.37 |  |
| ***UGT1A3*** | |  |  |  |  |  |  |  |
| Controls | | 1.00 | 0.57 | 57% | 0.79 | 0.29 | 2.39 | 0.001 |
| WD | | 0.27 | 0.19 | 70% | 0.20 | 0.02 | 0.64 |  |
| ***UGT2B7*** | |  |  |  |  |  |  |  |
| Controls | | 1.00 | 0.38 | 38% | 0.99 | 0.39 | 1.97 | 2*10^-4^ |
| WD | | 0.20 | 0.16 | 82% | 0.16 | 0.00 | 0.53 |  |
| ***UGT2B15*** | |  |  |  |  |  |  |  |
| Controls | | 1.00 | 0.51 | 51% | 0.88 | 0.32 | 2.72 | 0.056 |
| WD | | 0.65 | 0.45 | 69% | 0.57 | 0.28 | 1.69 |  |
| ***ABCB1*** | |  |  |  |  |  |  |  |
| Controls | | 1.00 | 0.37 | 37% | 0.96 | 0.37 | 1.73 | 0.038 |
| WD | | 2.10 | 1.44 | 69% | 1.91 | 0.70 | 5.31 |  |
| ***ABCB11*** | |  |  |  |  |  |  |  |
| Controls | | 1.00 | 0.28 | 28% | 1.03 | 0.36 | 1.44 | 0.019 |
| WD | | 0.56 | 0.39 | 70% | 0.42 | 0.07 | 1.22 |  |
| ***ABCC1*** | |  |  |  |  |  |  |  |
| Controls | | 1.00 | 0.35 | 35% | 0.92 | 0.55 | 2.22 | 2*10^-4^ |
| WD | | 4.04 | 1.92 | 48% | 3.84 | 1.92 | 8.21 |  |
| ***ABCC2*** | |  |  |  |  |  |  |  |
| Controls | | 1.00 | 0.33 | 33% | 0.86 | 0.58 | 1.80 | 0.081 |
| WD | | 0.68 | 0.45 | 66% | 0.73 | 0.13 | 1.61 |  |
| ***ABCC3*** | |  |  |  |  |  |  |  |
| Controls | | 1.00 | 0.35 | 35% | 0.94 | 0.42 | 1.85 | 0.103 |
| WD | | 1.50 | 0.58 | 38% | 1.79 | 0.76 | 2.21 |  |
| ***ABCC4*** | |  |  |  |  |  |  |  |
| Controls | | 1.00 | 0.56 | 56% | 0.86 | 0.50 | 3.12 | 1*10^-4^ |
| WD | | 8.46 | 4.50 | 53% | 6.43 | 3.83 | 18.39 |  |
| ***ABCG2*** | |  |  |  |  |  |  |  |
| Controls | | 1.00 | 0.46 | 46% | 0.98 | 0.28 | 2.37 | 0.001 |
| WD | | 0.36 | 0.25 | 70% | 0.27 | 0.10 | 0.95 |  |
| ***SLC10A1*** | |  |  |  |  |  |  |  |
| Controls | | 1.00 | 0.29 | 29% | 0.99 | 0.41 | 1.65 | 2*10^-4^ |
| WD | | 0.19 | 0.12 | 63% | 0.18 | 0.02 | 0.37 |  |
| ***SLC16A1*** | |  |  |  |  |  |  |  |
| Controls | | 1.00 | 0.46 | 46% | 1.04 | 0.24 | 2.35 | 3*10^-4^ |
| WD | | 0.19 | 0.13 | 66% | 0.13 | 0.04 | 0.45 |  |
| ***SLC22A1*** | |  |  |  |  |  |  |  |
| Controls | | 1.00 | 0.38 | 38% | 0.99 | 0.28 | 1.74 | 1*10-4 |
| WD | | 0.13 | 0.08 | 59% | 0.16 | 0.01 | 0.23 |  |
| ***SLC22A3*** | |  |  |  |  |  |  |  |
| Controls | | 1.00 | 0.36 | 36% | 0.90 | 0.54 | 1.89 | 0.017 |
| WD | | 0.57 | 0.37 | 64% | 0.42 | 0.25 | 1.27 |  |
| ***SLC22A7*** | |  |  |  |  |  |  |  |
| Controls | | 1.00 | 0.27 | 27% | 1.00 | 0.41 | 1.39 | 0.010 |
| WD | | 0.51 | 0.36 | 71% | 0.39 | 0.05 | 1.17 |  |
| ***SLC22A18*** | |  |  |  |  |  |  |  |
| Controls | | 1.00 | 0.19 | 19% | 0.93 | 0.65 | 1.38 | 0.006 |
| WD | | 1.64 | 0.65 | 40% | 1.52 | 0.89 | 3.09 |  |
| ***SLCO1B1*** | |  |  |  |  |  |  |  |
| Controls | | 1.00 | 0.34 | 34% | 0.97 | 0.47 | 1.82 | 1*10^-4^ |
| WD | | 0.22 | 0.11 | 50% | 0.23 | 0.02 | 0.35 |  |
| ***SLCO1B3*** | |  |  |  |  |  |  |  |
| Controls | | 1.00 | 0.40 | 40% | 1.05 | 0.24 | 2.19 | 0.072 |
| WD | | 0.58 | 0.66 | 113% | 0.39 | 0.04 | 1.92 |  |
| ***SLCO2B1*** | |  |  |  |  |  |  |  |
| Controls | | 1.00 | 0.24 | 24% | 0.96 | 0.61 | 1.64 | 4*10^-4^ |
| WD | | 0.52 | 0.20 | 39% | 0.50 | 0.14 | 0.80 |  |

Relative quantity (RQ) of each transcript presented in the table is normalized to mean value for the control group (ΔΔCT method); CV – coefficient of variation.

WD – Wilson’s disease, All p values obtained by non-parametric Mann-Whitney U test.
