# Supplementary material for: Hepatic drug-metabolizing enzymes and drug transporters in Wilson’s disease patients with liver failure
Source: Pharmacol Rep. 2021 Jun 11;73(5):1427–38. doi: 10.1007/s43440-021-00290-8 (PMC8460590; doi:10.1007/s43440-021-00290-8)
Supplement: Supplementary file 2 — Supplementary file2 (DOCX 24 kb) [file 43440_2021_290_MOESM2_ESM.docx]

Supplementary Table 3. Protein quantity data of the biotransformation enzymes and drug transporters in controls (n=20) and Wilson’s disease livers (n=7).

|  | mean | SD | CV% | median | min | max | positive | P value |
| --- | --- | --- | --- | --- | --- | --- | --- | --- |
| Protein  [fmol/mg] | | | | | | | |  |
| **CYP1A1** |  |  |  |  |  |  |  |  |
| Controls | 37.68 | 48.28 | 128% | 21.50 | 0.00 | 190.27 | 18/20 | 0.049 |
| WD | 10.73 | 8.91 | 83% | 10.56 | 0.00 | 28.13 | 5/7 |  |
| **CYP1A2** |  |  |  |  |  |  |  |  |
| Controls | 953.10 | 702.40 | 74% | 768.55 | 40.58 | 2645.59 | 20/20 | 0.019 |
| WD | 370.95 | 373.55 | 101% | 205.35 | 0.00 | 1084.95 | 6/7 |  |
| **CYP2B6** |  |  |  |  |  |  |  |  |
| Controls | 103.05 | 67.45 | 65% | 91.57 | 14.28 | 292.68 | 20/20 | 0.143 |
| WD | 71.29 | 89.73 | 126% | 43.31 | 0.00 | 271.55 | 5/7 |  |
| **CYP2C8** |  |  |  |  |  |  |  |  |
| Controls | 538.74 | 294.00 | 55% | 532.83 | 101.21 | 1136.74 | 20/20 | 0.005 |
| WD | 162.53 | 142.61 | 88% | 174.37 | 2.43 | 408.82 | 7/7 |  |
| **CYP2C9** |  |  |  |  |  |  |  |  |
| Controls | 2290.74 | 1213.74 | 53% | 2007.57 | 630.81 | 5676.82 | 20/20 | 0.014 |
| WD | 976.23 | 705.72 | 72% | 914.95 | 30.60 | 2260.73 | 7/7 |  |
| **CYP2C19** |  |  |  |  |  |  |  |  |
| Controls | 363.07 | 173.56 | 48% | 332.11 | 81.13 | 797.67 | 20/20 | 0.056 |
| WD | 640.01 | 474.44 | 74% | 432.88 | 347.10 | 1773.99 | 7/7 |  |
| **CYP2D6** |  |  |  |  |  |  |  |  |
| Controls | 346.43 | 234.97 | 68% | 291.30 | 0.00 | 852.82 | 19/20 | 0.347 |
| WD | 272.98 | 251.07 | 92% | 240.38 | 0.00 | 832.53 | 6/7 |  |
| **CYP2E1** |  |  |  |  |  |  |  |  |
| Controls | 1549.54 | 774.77 | 50% | 1394.06 | 272.07 | 3407.44 | 20/20 | 0.213 |
| WD | 1100.17 | 861.68 | 78% | 918.17 | 107.70 | 2328.62 | 7/7 |  |
| **CYP3A4** |  |  |  |  |  |  |  |  |
| Controls | 967.18 | 708.88 | 73% | 672.99 | 99.21 | 2634.15 | 20/20 | 0.012 |
| WD | 279.15 | 363.31 | 130% | 75.01 | 0.00 | 1051.19 | 4/7 |  |
| **CYP3A5** |  |  |  |  |  |  |  |  |
| Controls | 108.82 | 150.08 | 138% | 50.67 | 20.96 | 679.89 | 20/20 | 0.029 |
| WD | 72.92 | 146.02 | 200% | 21.11 | 0.00 | 429.19 | 5/7 |  |
| **UGT1A1** |  |  |  |  |  |  |  |  |
| Controls | 641.33 | 294.15 | 46% | 619.53 | 182.88 | 1123.72 | 20/20 | 0.391 |
| WD | 824.44 | 410.04 | 50% | 875.58 | 170.02 | 1484.78 | 7/7 |  |
| **UGT1A3** |  |  |  |  |  |  |  |  |
| Controls | 235.37 | 144.45 | 61% | 205.20 | 80.90 | 657.50 | 20/20 | 0.761 |
| WD | 220.87 | 130.67 | 59% | 156.79 | 89.06 | 487.78 | 7/7 |  |
| **UGT2B7** |  |  |  |  |  |  |  |  |
| Controls | 1904.59 | 868.55 | 46% | 1700.99 | 344.22 | 3455.79 | 20/20 | 0.092 |
| WD | 1168.07 | 1188.79 | 102% | 551.85 | 224.34 | 3047.98 | 7/7 |  |
| **UGT2B15** |  |  |  |  |  |  |  |  |
| Controls | 629.15 | 330.01 | 52% | 550.30 | 174.36 | 1670.44 | 20/20 | 0.092 |
| WD | 419.80 | 350.21 | 83% | 282.48 | 81.71 | 1197.51 | 7/7 |  |
| **P-gp** |  |  |  |  |  |  |  |  |
| Controls | 4.07 | 7.54 | 185% | 0.63 | 0.00 | 31.60 | 12/20 | 0.009 |
| WD | 22.33 | 15.40 | 69% | 15.01 | 0.00 | 43.30 | 6/7 |  |
| **BSEP** |  |  |  |  |  |  |  |  |
| Controls | 60.33 | 34.14 | 57% | 64.13 | 9.30 | 118.39 | 20/20 | 0.049 |
| WD | 28.87 | 31.11 | 108% | 23.09 | 0.00 | 84.02 | 4/7 |  |
| **MRP1** |  |  |  |  |  |  |  |  |
| Controls | 6.54 | 17.17 | 263% | 0.00 | 0.00 | 73.12 | 5/20 | 0.676 |
| WD | 9.38 | 22.99 | 245% | 0.00 | 0.00 | 65.69 | 1/7 |  |
| **MRP2** |  |  |  |  |  |  |  |  |
| Controls | 170.70 | 113.55 | 67% | 185.87 | 6.20 | 317.88 | 20/20 | 0.019 |
| WD | 23.13 | 11.47 | 50% | 27.85 | 7.54 | 40.27 | 7/7 |  |
| **MRP3** |  |  |  |  |  |  |  |  |
| Controls | 23.37 | 44.50 | 190% | 2.05 | 0.00 | 179.98 | 11/20 | 0.108 |
| WD | 16.58 | 10.78 | 65% | 14.36 | 3.03 | 31.31 | 7/7 |  |
| **MRP4** |  |  |  |  |  |  |  |  |
| Controls | 0.00 | 0.00 | - | 0.00 | 0.00 | 0.00 | 0/20 | 6*10^-6^ |
| WD | 2.36 | 1.80 | 76% | 1.97 | 0.00 | 5.08 | 6/7 |  |
| **BCRP** |  |  |  |  |  |  |  |  |
| Controls | 1.51 | 5.04 | 334% | 0.00 | 0.00 | 22.96 | 2/20 | 0.879 |
| WD | 0.49 | 1.20 | 245% | 0.00 | 0.00 | 3.42 | 1/7 |  |
| **NTCP** |  |  |  |  |  |  |  |  |
| Controls | 119.96 | 73.78 | 62% | 104.13 | 34.62 | 267.24 | 20/20 | 0.005 |
| WD | 33.11 | 41.98 | 127% | 17.17 | 0.00 | 122.51 | 5/7 |  |
| **MCT1** |  |  |  |  |  |  |  |  |
| Controls | 115.68 | 83.27 | 72% | 103.74 | 17.94 | 346.09 | 20/20 | 0.115 |
| WD | 59.14 | 38.58 | 65% | 63.66 | 1.13 | 109.65 | 7/7 |  |
| **OCT1** |  |  |  |  |  |  |  |  |
| Controls | 91.70 | 74.66 | 81% | 72.86 | 0.00 | 280.18 | 18/20 | 0.157 |
| WD | 53.58 | 68.42 | 128% | 32.66 | 0.00 | 205.73 | 4/7 |  |
| **OCT3** |  |  |  |  |  |  |  |  |
| Controls | 6.78 | 8.88 | 131% | 1.26 | 0.00 | 29.24 | 12/20 | 0.887 |
| WD | 3.68 | 4.00 | 109% | 2.80 | 0.00 | 11.74 | 5/7 |  |
| **OAT2** |  |  |  |  |  |  |  |  |
| Controls | 31.67 | 26.01 | 82% | 20.26 | 0.00 | 93.35 | 19/20 | 0.391 |
| WD | 25.31 | 30.17 | 119% | 12.22 | 1.27 | 92.40 | 5/7 |  |
| **IMPT1** |  |  |  |  |  |  |  |  |
| Controls | 89.27 | 96.13 | 108% | 26.78 | 0.00 | 381.48 | 19/20 | 0.638 |
| WD | 73.70 | 51.08 | 69% | 70.95 | 13.72 | 162.71 | 7/7 |  |
| **OATP1B1** |  |  |  |  |  |  |  |  |
| Controls | 360.73 | 198.77 | 55% | 328.73 | 72.66 | 645.32 | 20/20 | 0.064 |
| WD | 193.44 | 132.04 | 68% | 146.98 | 19.51 | 397.78 | 7/7 |  |
| **OATP1B3** |  |  |  |  |  |  |  |  |
| Controls | 63.94 | 49.00 | 77% | 57.71 | 0.00 | 177.97 | 19/20 | 0.489 |
| WD | 77.50 | 104.23 | 134% | 15.20 | 1.38 | 298.69 | 7/7 |  |
| **OATP2B1** |  |  |  |  |  |  |  |  |
| Controls | 114.62 | 92.77 | 81% | 86.90 | 16.50 | 355.47 | 20/20 | 0.009 |
| WD | 20.78 | 18.72 | 90% | 10.23 | 0.00 | 47.19 | 6/7 |  |

All results are given in fmol/mg of the analyzed tissue. CV - coefficient of variation.

WD – Wilson’s disease. All p values obtained by non-parametric Mann-Whitney U test.
