# Supplementary material for: Hepatic drug-metabolizing enzymes and drug transporters in Wilson’s disease patients with liver failure
Source: Pharmacol Rep. 2021 Jun 11;73(5):1427–38. doi: 10.1007/s43440-021-00290-8 (PMC8460590; doi:10.1007/s43440-021-00290-8)
Supplement: Supplementary file 3 — Supplementary file3 (DOCX 26 kb) [file 43440_2021_290_MOESM3_ESM.docx]

**Supplementary Table 1.** List of assays used for mRNA quantification

| **Gene symbol** | **Assay ID (Thermo Fisher Scientific)** |
| --- | --- |
| *CYP1A1* | Hs00153120_m1 |
| *CYP1A2* | Hs00167927_m1 |
| *CYP2B6* | Hs03044631_m1 |
| *CYP2C8* | Hs04183483_g1 |
| *CYP2C9* | Hs02383631_s1 |
| *CYP2C19* | Hs00426380_m1 |
| *CYP2D6* | Hs00164385_m1 |
| *CYP2E1* | Hs00559367_m1 |
| *CYP3A4* | Hs00604506_m1 |
| *CYP3A5* | Hs01070905_m1 |
| *UGT1A1* | Hs02511055_s1 |
| *UGT1A3* | Hs04194492_g1 |
| *UGT2B7* | Hs00426592_m1 |
| *UGT2B15* | Hs00870076_s1 |
| *ABCB1* | Hs00184500_m1 |
| *ABCB11* | Hs00184824_m1 |
| *ABCC1* | Hs01561502_m1 |
| *ABCC2* | Hs00166123_m1 |
| *ABCC3* | Hs00978473_m1 |
| *ABCC4* | Hs00988717_m1 |
| *ABCG2* | Hs01053790_m1 |
| *SLC10A1* | Hs00161820_m1 |
| *SLC16A1* | Hs01560299_m1 |
| *SLC22A1* | Hs00427552_m1 |
| *SLC22A3* | Hs00222691_m1 |
| *SLC22A7* | Hs00198527_m1 |
| *SLC22A18* | Hs00180039_m1 |
| *SLCO1B1* | Hs00272374_m1 |
| *SLCO1B3* | Hs00251986_m1 |
| *SLCO2B1* | Hs01030353_m1 |
| *GAPDH* | Hs99999905_m1 |
| *GUSB* | Hs00939627_m1 |
| *HMBS* | Hs00609297_m1 |
| *PPIA* | Hs99999904_m1 |
| *RPLP0* | Hs99999902_m1 |
| *RPS9* | Hs02339424_g1 |
